# Physician-Directed Heart Failure Transitional Care Program: A Retrospective Case Review

**DOI:** 10.4021/jocmr1492w

**Published:** 2013-08-05

**Authors:** Ken S. Ota, David S. Beutler, Richard D. Gerkin, Jessica L. Weiss, Akil I. Loli

**Affiliations:** a Banner Good Samaritan Medical Center, Department of Transitional Care Medicine, Phoenix, Arizona, USA; b Cardiology Fellowship Program, Phoenix, Arizona, USA; c Internal Medicine Residency Program, Phoenix, Arizona, USA

**Keywords:** Transitional care, Transitionalist, Heart failure, Readmission

## Abstract

**Background:**

Despite a variety of national efforts to improve transitions of care for patients at risk for rehospitalization, 30-day rehospitalization rates for patients with heart failure have remained largely unchanged.

**Methods:**

This is a retrospective review of 73 patients enrolled in our hospital-based, physican-directed Heart Failure Transitional Care Program (HFTCP). This study evaluated the 30- and 90- day readmission rates before and after enrollment in the program. The Transitionalist’s services focused on bedside consultation prior to hospital discharge, follow-up home visits within 72 hours of discharge, frequent follow-up phone calls, disease-specific education, outpatient intravenous diuretic therapy, and around-the-clock telephone access to the Transitionalist.

**Results:**

The pre-enrollment 30-day readmission rates for acute decompensated heart failure (ADHF) and all-cause readmission was 26.0% and 28.8%, respectively, while the post-enrollment rates for ADHF and all-cause readmission were 4.1% (P < 0.001) and 8.2% (P = 0.002), respectively. The pre-enrollment 90-day all-cause and ADHF readmission rates were 69.8%, and 58.9% respectively, while the post-enrollment rates for all-cause and ADHF were 27.3% (P < 0.001) and 16.4% (P < 0.001) respectively.

**Conclusions:**

Our physician-implemented HFTCP reduced rehospitalization risk for patients enrolled in the program. This program may serve as a model to assist other hospital systems to reduce readmission rates of patients with HF.

## Introduction

Over five million people suffer from heart failure (HF) in the United States [1]. As the population continues to age, HF has become an increased burden on the healthcare system [2]. This is largely in part due to the aggressive management of cardiac disease which enables these patients to live long enough to develop HF. The American Heart Association [3] reported that hospitalizations for HF have tripled over a 25 year period from 1979 to 2004 (1.27 million 3.86 million). In a study of Medicare claims data from 2003 to 2004, nearly 27% of patients discharged with a primary diagnosis of ADHF were readmitted within 30 days [4]. The majority of patients who present to the emergency department with ADHF are admitted for inpatient treatment [5, 6].

Gheorghiade and Peterson [7] concluded that since HF is a complex syndrome caused by different underlying cardiac pathologies, health care providers must identify specific and targeted therapies for each individual patient. Providers must also recognize and treat both cardiac and non-cardiac comorbidities that may lead to future events of acute HF decompensation. The authors recognize that certain home-based devices, such as blood pressure monitors, scales, and pulse oximeters, provide daily patient-based data to clinicians as a method to recognize decompensation early.

HF is also well known for its enormous economic impact on the healthcare system. A large portion of treatment cost arises from hospital admissions and readmissions. It is estimated that in 2013 the total cost of HF will be $32 billion and is projected to increase to $70 billion by 2030 [1]. A provision of the Patient Protection and Affordable Care Act states that in 2013 the Centers for Medicaid and Medicare Services (CMS) begins penalizing hospitals with greater-than-expected HF readmission rates by imposing reimbursement penalties [8]. These penalties make it imperative for hospitals to reassess clinical practice patterns and identify modifications necessary to prevent financial losses that would arise from reimbursement penalties.

Despite the initiation of the American College of Cardiology/American Heart Association (ACC/AHA) HF performance measures in 2005, 30-day readmission rates for HF patients have remained largely unchanged according to a study published in 2012 of 3,655 hospitals in the United States [9]. Documentation of left ventricular systolic function, use of angiotensin-converting enzyme inhibitors or angiotensin-receptor blockers for left ventricular systolic dysfunction, providing smoking cessation advice and counseling, and giving written discharge instructions have not significantly reduced readmission rates for HF patients. The financial burden of HF on the health care industry and the need to improve care for HF patients requires new strategies to reduce readmission rates for ADHF patients [10, 11].

The 2009 Focused Update of the ACC/AHA Guidelines for the Diagnosis and Management of Heart Failure in Adults established a new Class 1B recommendation that states “Post-discharge systems of care, if available, should be used to facilitate the transition to effective outpatient care for patients hospitalized with heart failure” [12]. To implement this recommendation and to improve patient care, Banner Good Samaritan Medical Center (BGSMC) in Phoenix, Arizona initiated the physician-directed Heart Failure Transitional Care Program (HFTCP or Program) [13, 14]. In this retrospective case series review of 73 patients enrolled in the HFTCP, the investigators evaluated the clinical outcomes arising from the program. The HFTCP uses a multipronged approach to decrease readmissions of ADHF patients. This Program, to our knowledge, is the only HF transitional care program directed and implemented by a physician that has been reported in the peer-reviewed literature in the United States.

## Methods

### Study design

This study is a retrospective case series of 73 enrolled patients at BGSMC from September 2, 2011 to September 25, 2012. Investigators performed a chart review of the Banner Health electronic medical record to compare the number and frequency of admissions of HF patients in the Banner Health system 90 days prior to enrollment in the HFTCP with the number and frequency of admissions 90 days post-enrollment. The HFTCP is operated by a single Transitionalist who is board certified in Family Medicine with special training in HF management.

### Population

BGSMC is one of the seven Banner Health hospitals in Arizona which serve about 40% of the nearly four million Maricopa County residents. BGSMC is a 650-bed tertiary referral center in downtown Phoenix, Arizona that serves a large number of indigent individuals. The hospital has eight residency programs along with nine fellowship training programs, including a cardiology fellowship program.

### Screening and enrollment

BGSMC educated its physicians and mid-level providers about the goals of the HFTCP through formal lectures and fliers on the telemetry floors. BGSMC encouraged its providers to identify patients with HF and refer them to the HFTCP. The HF Transitionalist met with patients prior to discharge to discuss the goals of the HFTCP and to assess their desire to participate. This study included only the patients who were enrolled in the Program for a minimum of 90 days.

### Inclusion criteria

Eligible patients included those with ADHF as their primary reason for hospitalization and lived within 15 miles of the hospital. A radius of 15 miles was chosen prior to the initiation of the Program to make multiple physician home visits on a given day realistic. Of the 80 patients who were referred to the program, 73 were enrolled. The seven patients who did not enroll after being referred were not interested in participating in the Program or met exclusion criteria. The enrollment period was from September 1, 2011 to September 25, 2012.

### Exclusion criteria

The Transitionalist evaluated all the patients for whom he received inpatient consults. Excluded patients included those who did not show interest in the Program, who showed evidence of active illicit drug abuse, or who had end-stage renal disease (ESRD) on hemodialysis. The ERSD patients were excluded because their volume status was primarily regulated with hemodialysis and not diuretics.

### Study conduct

The BGSMC Institutional Review Board approved this study. All Health Insurance Portability and Accountability Act regulations were followed.

### Intervention

After each enrolled patient was educated on the goals of the Program in the hospital, each patient received direct around-the-clock access to the Transitionalist via his cell phone. The Program had several key interventions designed to decrease the risk of rehospitalizations and to improve quality of life which are described in more detail below in the discussion section and in prior publications [13, 14].

### Outcomes

The primary outcome of the study was a comparison of 30- and 90-day readmission rates before and after enrollment in the HFTCP of both ADHF and non-ADHF readmissions. The secondary outcome was a comparison of the total number of 30- and 90-day hospitalizations with a primary discharge diagnosis of ADHF or non-ADHF with the number of 30- and 90-day hospitalizations after enrollment in the HFTCP.

The other outcomes and variables reported in this study are the following: mortality rate of patients enrolled in the Program (expected due to hospice referral versus unexpected), number of outpatient IV diuretic therapy sessions and whether or not any complications occurred, number of patients who discontinued the Program and their reasons for discontinuing the Program, and the number of patients referred to hospice. The BGSMC 30-day readmission rate reported on the hospital’s intranet system via the Banner Health Score Card was reviewed. The study compared the all-cause 30-day readmission rate 6 months prior to the inception of the HFTCP (March 1, 2011 to August 31, 2011) with the 30-day readmission rate after the Program began (Sept 1, 2011 to February 29, 2012).

### Data collection

Study information was recorded on Microsoft^®^ Office Excel 2010 spreadsheets. Demographic information, all hospitalization dates 90 days prior to index hospitalization and 90 days post-enrollment, insurance status, post-hospital disposition, NYHA class, height, weight, laboratory data, number of prescriptions at discharge and past medical history were obtained from a chart review of inpatient records in Cerner Millennium.

### Statistical analysis

SPSS 18.0 (SPSS, Inc., 2010) was used along with Microsoft^®^ Office Excel 2010 for data analysis. Continuous variables were reported as means and standard deviations. Categorical variables were reported as percentages. Paired continuous outcomes were analyzed using Wilcoxon signed ranks tests since these variables were not normally distributed. McNemar’s test was used for paired categorical outcomes. A two-tailed P < 0.05 was considered significant.

## Results

Of the 80 patients that were referred to the Program, 73 enrolled. For demographic information of the population in the study was shown in Table 1.

**Table 1:** Patient Demographics and Clinical Profiles

	Number	Percentage	Standard Deviation
Age	67		16
Gender			
Male	39	53%	
Female	34	47%	
Race			
Caucasian	41	56%	
African American	20	27%	
Hispanic	12	17%	
Insurance			
Commercial	27	37%	
Medicare	19	26%	
Medicaid	15	20.5%	
Medicare Advantage	7	9.5%	
Uninsured	5	7%	
Disposition			
Home	54	74%	
Home Hospice	10	14%	
Skilled Nursing Facility	5	7%	
Inpatient Hospice	3	4%	
Assisted Living	1	1%	
NYHA class			
Class II	29	40%	
Class III	27	37%	
Class IV	17	23%	
Average Class	2.8		0.8
Medical Information			
Obesity (BMI > 30)	36	49%	
Diabetes Mellitus type 2	42	58%	
Atrial Fibrillation	26	36%	
Discharge Information			
Creatinine at Discharge	1.35		0.76
Number of prescriptions	12.1		4.7
Hospital weight loss (kg)	4.78		5.9
Mortality rate			
Total	19	26%	
Expected	16	22%	
Unexpected	3	4%	
Number that discontinued the program	8	11%	

Table 2 shows the decrease in the percentage of patients that had at least one 30-day and 90-day readmission during the 90 day post-enrollment periods. The 30-day readmission rate for ADHF pre-enrollment was 26.0% and post-enrollment was 4.1% (P < 0.001), and for all-cause 30-day readmission rate pre-enrollment was 28.8% while the post-enrollment rate was 8.2% (P = 0.002). The 90-day readmission rate for ADHF pre-enrollment was 58.9% and the post-enrollment rate was 16.4% (P < 0.001). All-cause 90-day readmission rate pre-enrollment was 69.8% while the post-enrollment rate was 27.4% (P < 0.001).

**Table 2:** Pre- and Post-Readmission Rates at 30 and 90 Days

	Pre-enrollment	Post-enrollment	P value
30-day ADHF readmission rate	26.0%	4.1%	< 0.001
30-day all cause readmission rate	28.8%	8.2%	0.002
90-day ADHF readmission rate	58.9%	16.4%	< 0.001
90-day all cause readmission rate	69.8%	27.3%	< 0.001

**Figure 1:**
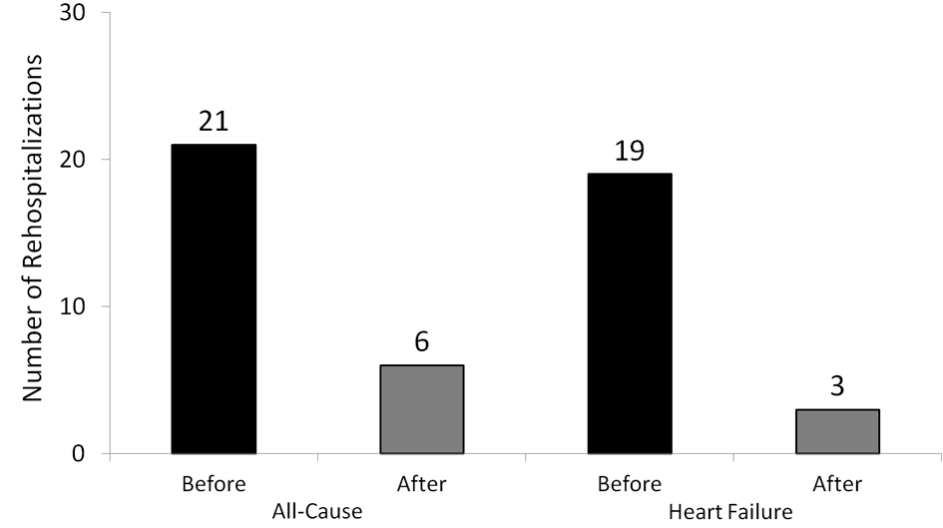
This graph shows the dramatic difference between the total number of 30-day acute decompensated heart failure and all cause readmissions of patients prior to enrollment compared with the total number of readmissions post-enrolment.

**Figure 2:**
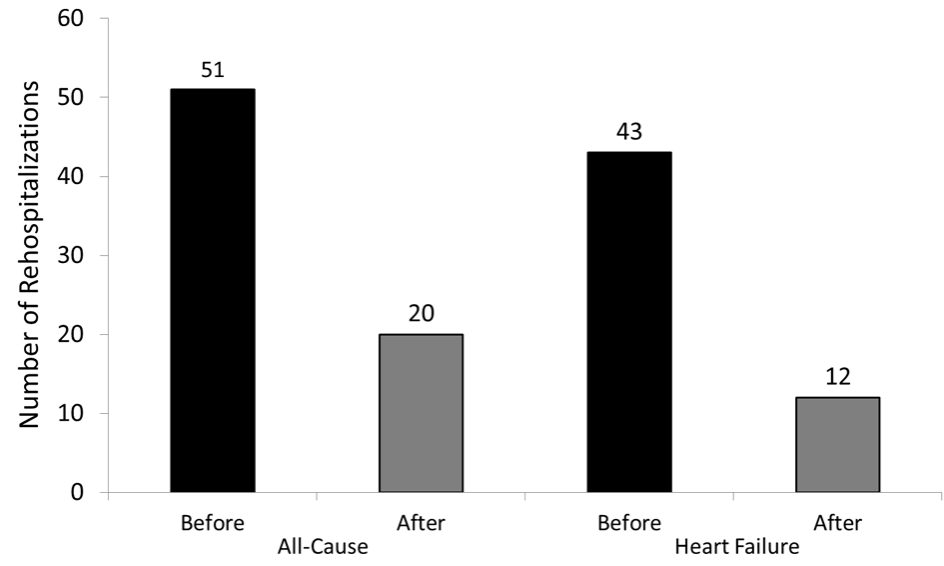
This graph shows the dramatic difference between the total number of 90-day acute decompensated heart failure and all cause readmissions of patients prior to enrollment compared with the total number of readmissions post-enrolment.

A difference between the total number of 30-day ADHF readmissions of patients prior to enrollment, 19, compared with 3 in the post enrollment period was observed (84% reduction) (Fig. 1). The number of 30-day readmissions for any cause was 21 in the pre-enrollment period compared with 6 post-enrollment (71% reduction). The number of 90-day readmissions for ADHF during the pre- and post-enrollment period were 43 and 12, respectively, (72% reduction) while there were 51 all-cause readmissions pre-enrollment and 20 readmissions post-enrollment (61% reduction) (Fig. 2). The average NYHA class was 2.8 at the time of discharge from the index hospitalization.

As a comparison, using the reported BGSMC HF readmission data available on the hospital’s intranet system, from March 1, 2011 to August 31, 2011 (prior to the inception of the HFTCP) the all-cause readmission rate for HF patients was 20.9% (17.7% with the study patients removed). From September 1, 2011 to February 29, 2012 (6 months after inception of the HFTCP), the readmission rate was 17.9% (15.9% with the study patients removed). Finally, from March 1, 2012 to August 31, 2012 (12 months after inception of the HFTCP), the readmission rate was 15.4% (14.1% with the study patients removed). The overall improvement in HF readmissions in non-study patients from before the study started until one year after was (17.7 - 14.1)/17.7 = 20.3%. This was likely due to other ongoing efforts by the hospital to reduce readmission rates. The baseline rate for study patients of readmission was 28.8%, indicating that those in the Program had a history of more frequent readmissions than those BGSMC HF patients not in the Program. The overall reduction in 30-day rehospitalization in the HFTCP patients ((28.8 - 8.2)/28.8 = 71.5%) was much greater than the 20.3% noted in other BGSMC patients.

The total mortality rate of the patients enrolled in the Program was 26% or 19 total deaths within 90 days post-enrollment. Of the 19 deaths, 16 patients had been referred to hospice care (because no meaningful recovery was expected) and 3 deaths were unexpected. Of the 16 patients referred to hospice only 13 agreed to hospice enrollment. None of the hospice-enrolled patients were readmitted to the hospital during the 90 days post-enrollment.

Ten total patients received outpatient IV diuretic therapy. No complications from outpatient IV diuretic therapy were reported [14, 15].

Eight of 73 (11%) patients were terminated from the Program after enrollment. These patients are included in all of the statistical analyses. Reasons for discontinuing the Program included lack of patient participation, inability to perform follow-up visits, continued illicit drug use, or self-discharge.

## Discussion

For a progressive disease, any reduction in the frequency of hospitalizations for ADHF is difficult to achieve, and breaking the demonstrated pattern of rehospitalization is challenging. Despite the implementation at BGSMC of the above-mentioned ACC/AHA HF performance measures, the 30-day readmission rate for HF patients has remained stagnant for the past several years. After implementing the physician directed HFTCP, the enrolled patients showed a rehospitalization rate significantly below historical average rates. When analyzing overall rehospitalization data for BGSMC, it appears that there is a trend toward a decreased readmission rate in the HF population since the inception of the Program.

Many published studies discuss the importance of post-discharge follow-up care for HF patients [16-19]. A pilot study at Baylor Medical Center Garland evaluated the effectiveness of a transitional care program for HF patients lead by an advanced practice nurse [20]. The Baylor study examined the effectiveness of its transitional care program on the “30-day all-cause readmission rate, length of stay and 60-day (from admission) direct cost” and reported a 48% reduction in adjusted readmission rate.

The results from this Baylor Study in conjunction with our study demonstrate that organized post-discharge systems of patient care are effective in reducing the 30-day readmission rates in the HF population. Interestingly, for reasons unclear to our group, seven of the 80 patients (8.8%) referred to the HFTCP were not interested in enrolling (one patient met exclusion criteria). In the Baylor study [18], 60% of the patients eligible for the transitional care intervention were not enrolled. The reasons for which patients referred to transitional care programs do not enroll should be investigated.

Our study shows dramatic decreases in both the rate and number of 30- and 90-day readmissions. Several key factors contributed to the successful reduction of hospital readmissions for patients enrolled in BGSMC’s HFTCP program. Our Transitionalist built trust with patients by first meeting them in the hospital prior to discharge and then setting up follow-up appointments within 72 hours of discharge at either the Transitionalist’s office, the patient’s home, or the patient’s skilled nursing facility. During the home visits, in addition to standard medical surveillance and follow-up, the Transitionalist provided dietary education and assisted each patient to evaluate the food in his or her cupboards to identify foods containing high levels of sodium. These visits were followed by phone calls to each enrolled patient from one to five times a week during the first 30 days after hospital discharge. The frequency of follow-up calls depended on the perceived need by the patient and the Transitionalist. Patients were encouraged to call the Transitionalist immediately if they experienced new or concerning symptoms. The Transitionalist was available around-the-clock via his cell phone. As a board certified Family Medicine physician, the Transitionalist helped manage other comorbidities along with the patients’ PCPs and specialists when necessary to help prevent return visits to the emergency department (ED) arising from even non-cardiac conditions.

By helping patients understand their hospital usage patterns, the Transitionalist provided patient-specific HF management education to identify ways to break frequent readmission cycles. With some patients, the Transitionalist used IV bolus diuretic therapy in the outpatient BGSMC infusion center to avoid unnecessary ED visits and subsequent hospitalizations [14]. The Transitionalist worked with patients, their families, and their community physicians to make appropriate referrals to hospice when necessary (for example, patients with end-stage HF who were not candidates for transplant or did not desire left ventricular assist devices). He continued to follow patients in hospice care, along with the other standard hospice providers. In conjunction with hospice providers, the Transitionalist helped keep all thirteen patients under hospice care from returning to the hospital. This model of individualized and hands-on care is an overwhelming task for the majority of PCPs and specialists who are unable to provide this type of around-the-clock care.

The Transitionalist was a salaried employee of the Banner Health system. Enrolled patients were not billed for follow-up visits with the Transitionalist to avoid patients declining to participate in the Program due to financial concerns. However, patients were required to make co-payments for the outpatient infusion center and observation unit stays.

The Program at BGSMC is an ongoing effort with over 190 patients that have enrolled since the end date of this study. Future publications will analyze the successes of the Program to provide a longer-term perspective. The hospital plans to grow the Program to include more providers, enroll a larger percentage of the HF patients at BGSMC, and implement the successful interventions of other TCPs.

### Study limitations

Though we recognize the benefit of our HFTCP, this study is not without limitations. First, only data from the Banner Health system was included in the study. It is conceivable that patients were readmitted to different hospital systems during the study period and did not report that information to the Transitionalist. We feel certain these patients were not readmitted elsewhere during the first 30 days after discharge, because the Transitionalist maintained close contact with these patients during the post-hospitalization period. Also we do not have readmission data from other hospital systems from the patients prior to enrollment into the Program, so it is conceivable that the pre-enrollment readmission rate was even higher than we reported. Second, a single Transitionalist managed all of the telephone calls throughout the entire study period, a task which may be too time-consuming for most physicians. The purpose of this clinical endeavor with a single physician was to merely demonstrate the efficacy of this physician-directed and implemented program. Third, individuals with ESRD on hemodialysis and/or with active illicit drug abuse history were excluded from the Program. We recognize that our goal should be to maximize the equity in healthcare delivery as much as possible. These patients were not included as we feel that they have special needs that could not be accommodated at the time of the implementation of this Program in its pilot stage. Fourth, in this study we did not do an in-depth cost-analysis of the Program. A cost-saving analysis will come forth in a future communication. In this study, we aimed to primarily demonstrate the efficacy of the HFTCP in reducing readmissions for the HF population. Finally, this is a retrospective case review study with only 73 patients. We recognize that a randomized control trial would provide a higher level of data. We plan to continually update our findings in future publications.

### Conclusion

The HFTCP at BGSMC succeeded in reducing the number of rehospitalizations at 30 and 90 days after discharge both for ADHF and for all causes. This Program may serve as a model to assist other hospital systems to reduce readmission rates of HF patients.

## Abbreviations

HF: Heart Failure
ADHF: Acute Decompensated Heart Failure
ACC/AHA: American College of Cardiology/American Heart Association
CMS: Centers for Medicaid and Medicare Services
PCPs: Primary Care Physicians
BGSMC: Banner Good Samaritan Medical Center
HFTCP or Program: Heart Failure Transitional Care Program
ESRD: End-stage Renal Disease
IV: Intravenous
NYHA: New York Heart Association
